# Perforation of anastomotic peptic ulcer following pancreaticoduodenectomy: a report of three cases

**DOI:** 10.1186/s12893-020-00743-6

**Published:** 2020-04-19

**Authors:** Ikuma Shioi, Naoyuki Yokoyama, Motoharu Hirai, Masaru Komatsu, Akira Kubota, Makoto Aoki, Daisuke Sato, Tetsuya Otani

**Affiliations:** Department of Digestive Surgery, Niigata City General Hospital, 463-7 Shumoku, Chou-ku, Niigata City, 950-1197 Japan

**Keywords:** Peptic ulcer, Gastroduodenal ulcer, Intestinal perforation, Gastric acid, Pancreatic juice, Pancreaticoduodenectomy, Surgery, Postoperative complication

## Abstract

**Background:**

Perforation of a marginal peptic ulcer after pancreaticoduodenectomy (PD) can lead to severe conditions, although its clinical features have not been well reported. In this article, we present three cases of marginal peptic ulcer perforation after PD that we experienced in our institute and attempt to clarify its appropriate treatment and prevention.

**Case presentation:**

Marginal ulcer perforation confirmed with computed tomography and/or surgical exploration occurred in 3 (1.8%) of 163 consecutive patients who underwent PD (including 160 patients who underwent a total or subtotal stomach-preserving procedure) at our institution. The three patients (one man and two women) had a median age of 77 (65–79) years. Two of these patients had a medical history of duodenal peptic ulcer. All three patients had biliary neoplasms. Two of the patients underwent subtotal stomach-preserving PD with antro-jejunal anastomosis, and the other patient underwent pylorus-preserving PD with duodenal jejunostomy. The perforation occurred with a sudden and severe onset of abdominal pain 34, 94, and 1204 days, respectively, after the PDs. At the time of the perforation, all of the patients had been withdrawn from postoperative prophylactic antipeptic ulcer agents, with the cessation periods ranging from 12 to 1008 days. In addition, all the patients were in fasting conditions for 1 to 13 days just before the perforation. Surgical treatment with direct suturing of the perforated ulcer was performed for two patients, while conservative therapy was performed for one patient. Their primary treatment courses were satisfactory. Chronic antisecretory agent therapy was prescribed for 562, 271, and 2370 days, respectively, from marginal ulcer perforation, and no ulcer recurrence was noted in any of the patients.

**Conclusions:**

Lack of antisecretory therapy and fasting were considered an essential cause of marginal peptic ulcer perforation after PD. In addition, unlike the native duodenum, the jejunal limb used for reconstruction to a preserved stomach may be at increased risk of ulceration. Chronic permanent administration of antisecretory agents and fasting avoidance are desirable for patients who have undergone stomach-preserving PD to prevent marginal ulcer perforation.

## Background

Pancreaticoduodenectomy (PD) is one of the most complex abdominal surgeries and is associated with high morbidity [1–3]. Anastomotic marginal peptic ulcer at a gastro/duodeno-enterostomy site is a well-known major complication after PD that occasionally leads to serious conditions. Although earlier studies have documented the incidence and pathophysiology of marginal ulcer, intestinal perforation owing to the disease has not been well discussed [4–6]. We report cases of three patients who suffered from perforation of a marginal ulcer after a stomach-preserving PD. By analyzing their clinical features, this article aims to clarify the appropriate treatment and prevention of marginal ulcer perforation.

### Case presentations

From January 2008 to December 2017, 163 patients underwent PD, including 160 patients who underwent a total or subtotal stomach-preserving procedure, in our institution. Of the 163 patients, 75 patients (44.7%) were diagnosed with biliary cancer, 63 (38.7%) with pancreatic cancer, and 26 (16.0%) with other diseases, such as intraductal papillary mucinous neoplasm, based on their clinical findings. Among them, three patients (1.8%), who underwent stomach-preserving PD for biliary cancer, developed marginal ulcer perforation.

### Case 1

**Fig. 1:**
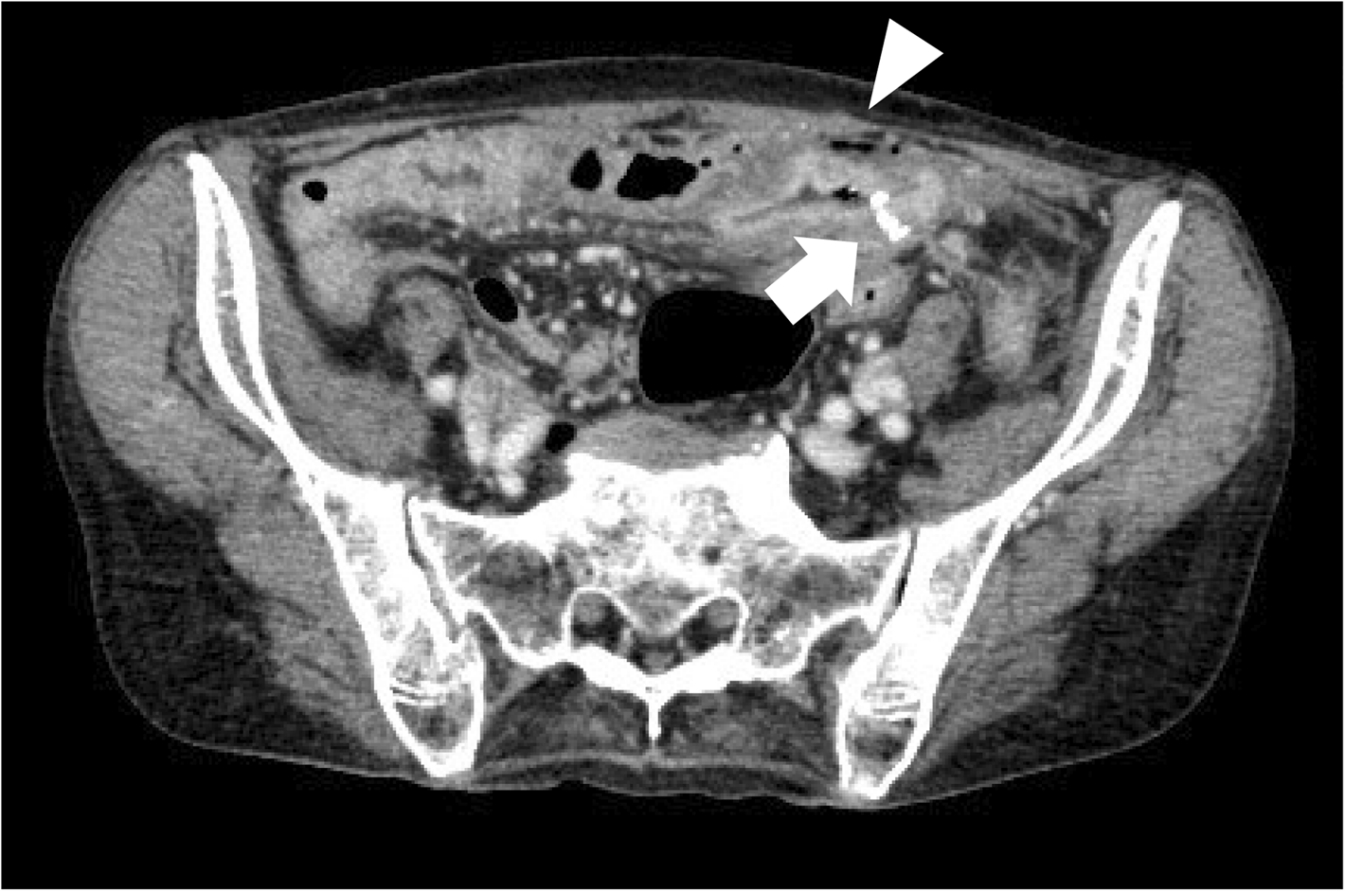
Computed tomography revealed intestinal edema at the duodenojejunostomy site where linear staplers were used (arrow). Free intraabdominal air was observed near the anastomosis site (arrowhead)

**Fig. 2:**
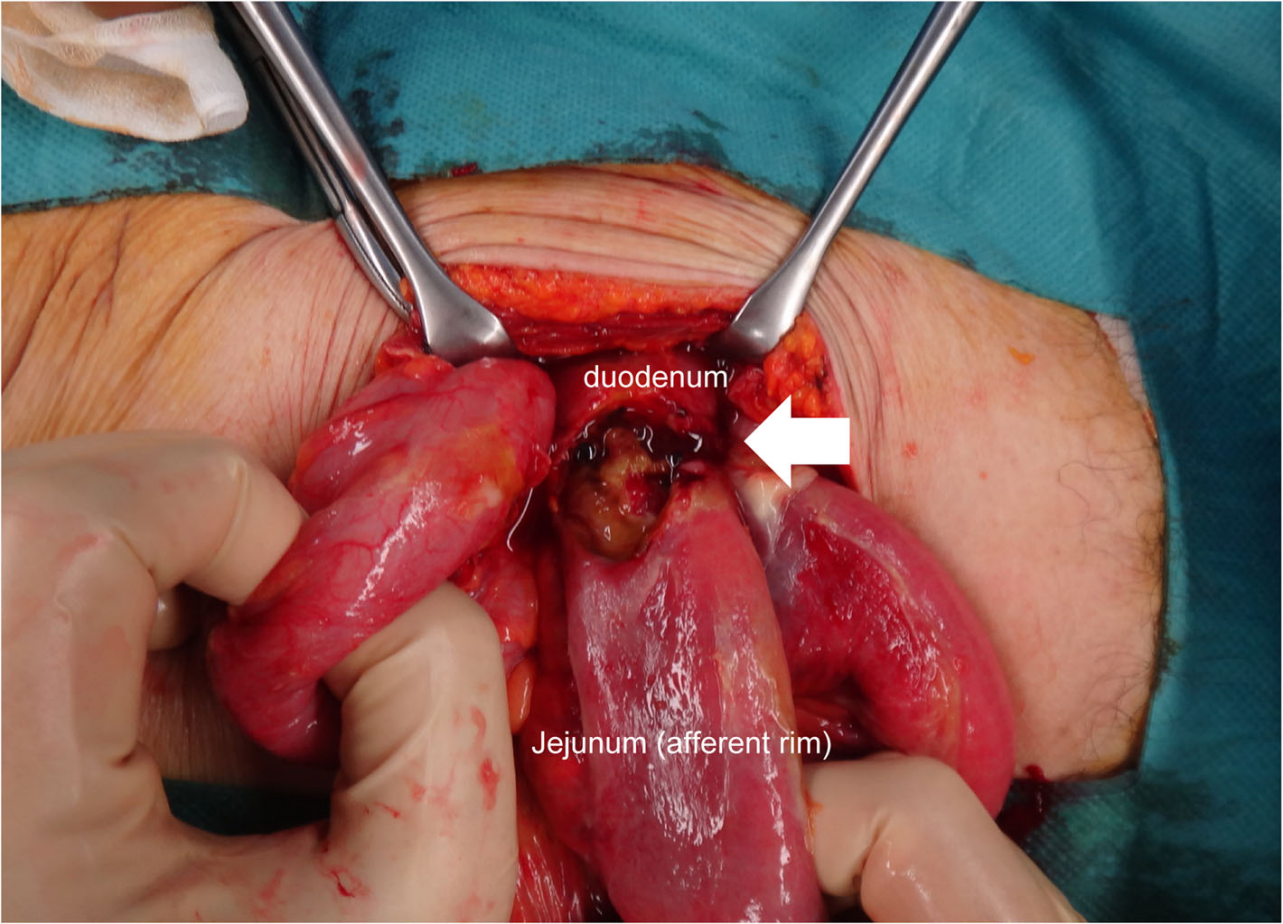
Marginal ulcer perforation was found at the jejunal side of the duodenojejunal anastomosis (arrow)

A 79-year-old woman without a medical history of peptic ulcer was diagnosed with duodenum papilla carcinoma. Pylorus-preserving PD was performed, and end-to-side duodenojejunostomy with the modified Child’s method (the enterostomy was distal to the pancreaticojejunostomy) was selected for intestinal reconstruction. After the surgery, a proton pump inhibitor (PPI) was administered for acid suppression, and she was discharged from the hospital on postoperative day (POD) 22. PPI therapy was withdrawn on the day of discharge. However, she experienced loss of appetite and often missed meals after returning home. Twelve days after discharge (POD 34), she experienced acute abdominal pain and presented at our emergency room. A tender abdomen was noted on physical assessment, and computed tomography (CT) revealed intestinal perforation at the duodenojejunal anastomosis site (Fig. 1). Surgical intervention was performed, and a marginal ulcer perforation was noted at the duodenojejunal anastomosis site (Fig. 2). Primary suture closure of the perforation site was performed, and omental patching was added without intestinal resection. Chronic antisecretory therapy was then prescribed for the patient. She gradually regained appetite and was discharged 28 days after the second surgery. The marginal ulcer was confirmed by a follow-up endoscopy. She died of intraabdominal cancer relapse on POD 596 after the initial surgery; however, the symptomatic marginal ulcer did not recur under chronic antisecretory therapy until her death.

### Case 2

A 76-year-old man who had a past history of non-steroidal anti-inflammatory drugs (NSAIDs)-induced ulcer was diagnosed with distal bile duct carcinoma. He underwent subtotal stomach-preserving PD and end-to-side gastrojejunostomy with the modified Child’s reconstruction. He was discharged a month after the surgery, and PPI was prescribed for 15 months and was withdrawn. Thirty-one months after the surgery, he was admitted to another hospital for a femoral fracture. To resolve the fracture pain, NSAIDs were used, but antisecretory agents were not prescribed. He gradually lost appetite and required fluid infusion during the hospitalization. Two months after the admission (2 weeks after starting fluid infusion), he experienced acute abdominal pain and was referred to our hospital. We observed a tender abdomen on physical assessment, and CT revealed intestinal perforation near the anastomosis site. We performed emergency laparotomy and detected a marginal ulcer perforation at the gastrojejunal anastomosis site. Primary closure of the perforation site with omental patching was performed. The patient was discharged 22 days after the emergency surgery. Chronic antisecretory agent was prescribed, and no recurrence of the ulcer occurred before he died of cancer relapse on POD 1195 after the initial surgery.

### Case 3

**Fig. 3:**
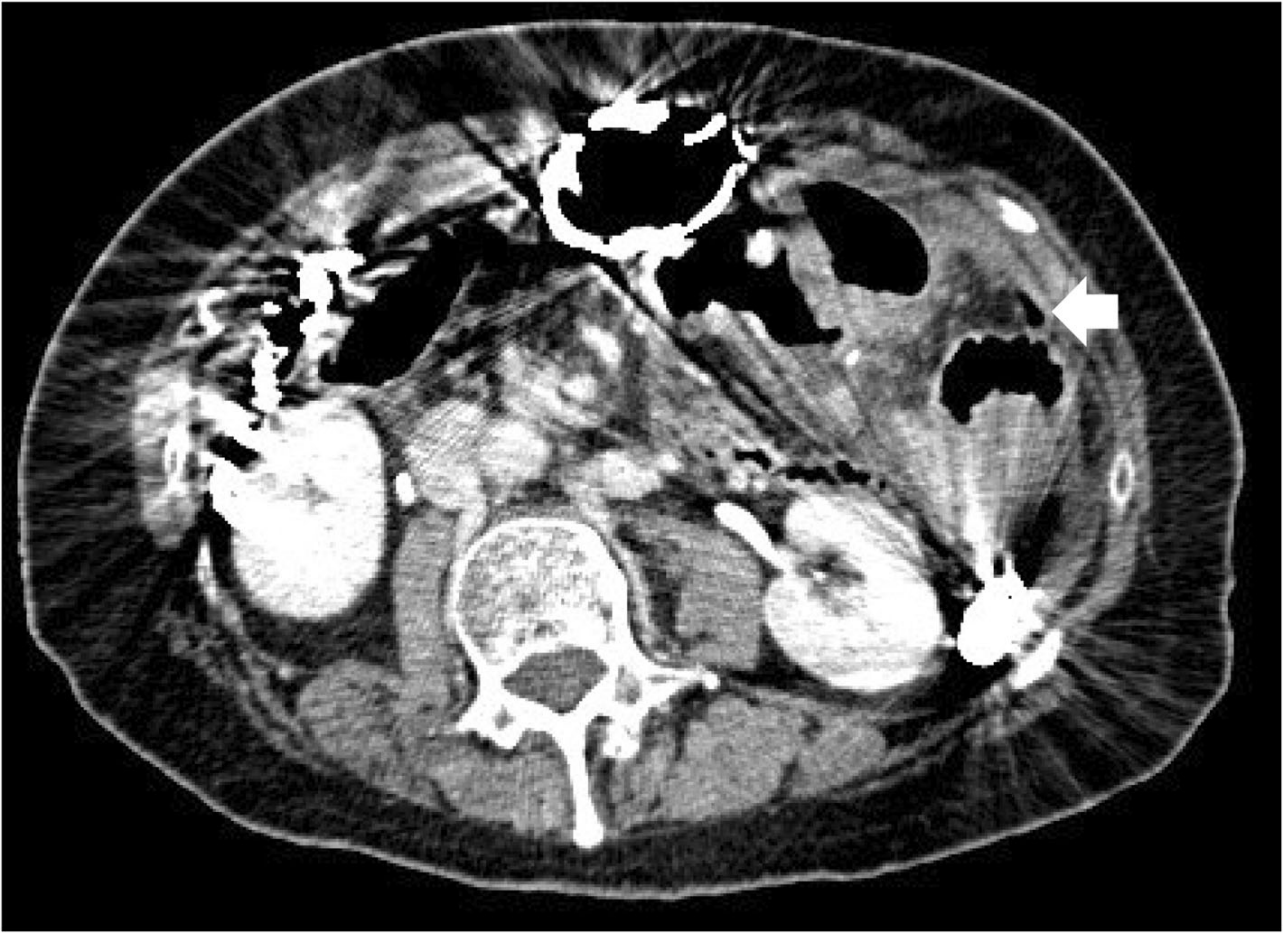
Computed tomography revealed free intraabdominal air near the anastomosis site (arrow)

A 62-year-old woman was diagnosed with distal bile duct carcinoma and underwent pylorus-preserving PD with end-to-side duodenojejunostomy using the modified Child method. Ulceration at the duodenum bulb was also identified during the preoperative examination. The postoperative course was uneventful. PPI was prescribed for 7 months and discontinued. Forty-three months after the surgery, she complained of hematochezia, and medical checkup with barium enema was scheduled. She needed to fast for a day before the examination. After the examination, she experienced acute abdominal pain. CT showed intraabdominal free air near the duodenojejunostomy anastomotic site, suggesting marginal ulcer perforation (Fig. 3). As her symptoms were relatively mild, she was treated conservatively with gastric drainage using nasogastric intubation and PPI administration. She was discharged 2 weeks after the admission without complications. She was placed on chronic antisecretory therapy, and to date, no ulcer or cancer recurrence has occurred.

## Discussion and conclusions

Marginal peptic ulcer at a gastro- or duodenojejunal anastomosis is a well-known complication after PD [7]. A recent study reported that the incidence of marginal ulcer after PD was 2.4%, occurring at a median time of 15.5 months after surgery, although the frequency of perforation was not demonstrated [1]. As presented in the current article, marginal ulcer perforation developed in 1.8% of the PD cases examined at our institution, which seemed non-negligible. The three presented cases had several common features, such as biliary malignant disease as the original disease, lack of antisecretory drug administration, and loss of appetite or a fasting condition, before the perforation event. By reviewing their clinical courses, this report might clarify the pathophysiology of marginal ulcer perforation after PD and aid the prevention and treatment of the disease.

The etiology of marginal peptic ulcer after PD is considered to be related to the altered gastrointestinal anatomy along with other conventional causes, such as gastric acidity, NSAID therapy, and Helicobacter pylori infection [5, 8–11]. Several acid-inhibiting hormones, such as secretin, gastric inhibitory polypeptide, and vasoactive intestinal peptide, are mainly produced in the small bowel, especially near the stomach [5]. In animal experiments comparing duodenum resection and non-resection [5], the resection group had 5.7 and 3.6 times more gastric acid secretion under fasting and diet conditions, respectively. Therefore, the removal of the duodenum and upper jejunum may accelerate the production of gastric acid. In addition, while the duodenum is rich in Brunner’s glands (which has alkaline-rich mucinous secretion), the jejunum limb used for reconstruction after PD lacks this gland. Therefore, the jejunum limb may not adequately neutralize gastric acid, leading to further ulcerogenic conditions [12]. As all the three presented cases of marginal peptic ulcer occurred after stomach-preserving surgery, that retained the fundamental secretion of gastric acid, it can be said that the anastomsed jejunum limb is at high risk of ulceration.

The time of emergence of ulcer perforation after the primary surgery varied between the three patients, ranging from 1 month to 3 years. Moreover, the cessation periods of antisecretory drug administration differed considerably between the three cases. However, their fasting durations were relatively short, ranging from 1 day to 2 weeks. These findings suggest that fasting promptly affect the development of marginal ulcer perforation, although this hypothesis needs further investigation [13–15]. Furthermore, the incidence of marginal ulcer perforation differs from that of the original disease, as all three cases occurred after PD for biliary cancer, accounting for 4% of the 75 cases. Unlike patients with pancreatic cancer, patients with biliary cancer often have preserved pancreatic secretory function; therefore, the neutralization of gastric acid should be more efficient in these patients. The reason for the paradoxical findings also remains uncertain and should be determined by the accumulation of case series.

Two of the three presented patients with marginal ulcer perforation were treated surgically via direct closure of the lesion, while conservative therapy was selected for the remaining patient. The patients’ post-treatment courses were substantially favorable. We believe that since all of them were fasting at the time of the perforation event, intraabdominal contamination was limited and severe infectious complications were avoided. In addition, chronic administration of antisecretory agents is considered useful for the prevention of ulcer recurrence [11]. The patients’ clinical courses suggest that the management strategy for marginal ulcer perforation might conform to that for conventional gastroduodenal peptic ulcer perforation. The patients in this report were not adequately evaluated for *H. pylori* infection except for one patient (Case 3, who was free of *H. pylori*). Further study concerning *H. pylori* involvement in marginal ulcer perforation after PD appears useful.

In conclusion, we presented three patients who experienced marginal ulcer perforation after PD. Exposure to excess acid due to the lack of antisecretory agent use and fasting was considered the essential cause of the marginal peptic ulcer perforation after PD. Chronic long-term administration of antisecretory agents and the avoidance of fasting are needed for patients who have undergone stomach-preserving PD, especially in cases of biliary disease or a history of peptic ulcer, to prevent marginal ulcer perforation.

## Abbreviations

CT: Computed tomography
NSAIDs: Non-steroidal anti-inflammatory drugs
PD: Pancreaticoduodenectomy
POD: Postoperative day
PPI: Proton pump inhibitor
